# Uma Colaboração para Parar de Fumar

**DOI:** 10.36660/abc.20200458

**Published:** 2021-01-27

**Authors:** Ricardo Vivacqua Cardoso Costa

**Affiliations:** 1 Hospital Pró-Cardíaco Rio de JaneiroRJ Brasil Hospital Pró-Cardíaco, Rio de Janeiro, RJ – Brasil

**Keywords:** Hábito de Fumar, Estilo de Vida Sedentario, Morbimortaliadade, Exercício, Sono, Sistema Nervoso Autônomo

A associação de tabagismo e sedentarismo constitui uma importante expressão de morbimortalidade em amplas faixas etárias.^1^

O presente estudo^2^ potencializa os reconhecidos riscos do tabagismo, correlacionando à qualidade do sono e às disfunções autonômicas com níveis de atividade física. Parâmetros considerados como fatores de risco para patologias cardiovasculares.^3^ Vale destacar à acurada metodologia contribuindo com a legitimação desse estudo. No grupo mais ativo (p>50) foram observados valores mais elevados da massa muscular esquelética, relacionados com o melhor desempenho e influenciando no metabolo reflexo.^4^ O tratamento estatístico demonstrou correlações significativas entre o fumo, a qualidade do sono e a disfunção autonômica. Acreditamos que um número mais elevado dos candidatos ao estudo, possa obter correlações mais fortes. Sugerimos como continuação desse estudo, avaliar a recuperação da frequência cardíaca no primeiro minuto da recuperação pós esforço, indicador de adaptação autonômica parassimpática de valor prognóstico nas afecções cardiovasculares.^5^ Vale, também, melhor quantificação de atividade física que caracteriza o comportamento ativo ou não.^6^ Merece destaque a bibliografia atual, com cerca de 16% de autores do nosso país.

Reiteramos a originalidade, delineamento e conclusões desse estudo,^2^ com ampla aplicabilidade prática, quando demonstra que o fumante ativo exibe parâmetros favoráveis relacionados à qualidade do sono e à disautonomia, podendo colaborar com a interrupção do hábito de fumar.
