# Supplementary material for: Uma Colaboração para Parar de Fumar
Source: Arq Bras Cardiol. 2021 Jan 27;116(1):36. [Article in Portuguese] doi: 10.36660/abc.20200458 (PMC8159517; doi:10.36660/abc.20200458)
Supplement: Translation

# Translation: A Collaboration to Stop Smoking

**Authors:** Ricardo Vivacqua Cardoso Costa

**Affiliations:** 1 Brazil Hospital Pró-Cardíaco, Rio de Janeiro, RJ – Brazil

**Keywords:** Atrial Fibrillation/physiopathology, Arrhythmias Cardiac/physiopathology, Risk Factors, Obesity, Sedentarism, Combined Modality Therapy

The association between smoking and physical inactivity is an essential expression of morbidity and mortality in wide age groups.^1^

The present study^2^enhances the recognized risks of smoking, correlating it to the quality of sleep, and autonomic dysfunctions with levels of physical activity. Parameters considered as risk factors for cardiovascular pathologies.^3^It is worth mentioning the accurate methodology contributing to the legitimacy of this study. In the most active group (p> 50), higher values of skeletal muscle mass were observed, related to better performance and influencing the reflex metabolite.^4^Statistical treatment demonstrated significant correlations between smoking, sleep quality, and autonomic dysfunction. We believe that a higher number of candidates for the study can obtain stronger associations. We suggest, as a continuation of this study, to evaluate the recovery of heart rate in the first minute of post-exercise recovery, an indicator of parasympathetic autonomic adaptation of prognostic value in cardiovascular diseases.^5^Also, a better quantification of physical activity that characterizes active or non-active behavior is also valid.^6^The current bibliography deserves mention, with about 16% of authors from our country.

We reiterate the originality, design, and conclusions of this study,^2^with broad practical applicability, when it demonstrates that the active smoker exhibits favorable parameters related to the quality of sleep and dysautonomia, being able to collaborate with the interruption of the smoking habit.
